# Does the use of intraoperative measurement reduce limb length discrepancies after total hip arthroplasty?

**DOI:** 10.1186/s12891-023-06774-3

**Published:** 2023-08-12

**Authors:** Junzhe Wu, Xunrong Zhuang, Chaohui Lin, Lijiang He, Rongmou Zhang

**Affiliations:** Department of Orthopaedic Surgery, The Second Affiliated Hospital, Fujian Medical University, Quanzhou, Fujian 362000 China

**Keywords:** Total hip arthroplasty, Limb length discrepancy, Retrospective study, Calcar femorale, Lower extremity length

## Abstract

**Purpose:**

Postoperative limb length discrepancy (LLD) is a common complication of total hip arthroplasty, and several methods exist to prevent LLD, but each has its benefits and drawbacks. The study investigates the application of intraoperative lower limb length measurement in preventing postoperative LLD.

**Methods:**

This study retrospectively analyzed 70 patients who underwent total hip arthroplasty from October 2018 to July 2022. The length of the lower limb on the operated side was measured intraoperatively using a sterilized paper ruler after the fitting of the trial mould and compared with the healthy side. Then the prosthesis size, depth and neck length were adjusted accordingly.

**Results:**

The absolute value of postoperative LLD was found to be 6.68 ± 4.48 mm, of which 53 cases (75.7%) were less than or equal to 10 mm, while 30 patients (42.9%) were less than or equal to 5 mm.

**Conclusion:**

The use of intraoperative measurement is effective in reducing LLD after total hip arthroplasty.

## Introduction

As the population ages, hip pain has seriously affected the quality of life of older adults, including femoral neck fracture, osteoarthritis, femoral head necrosis, and acetabular dysplasia. Total Hip Arthroplasty (THA) technology is well-established and is currently the primary treatment for hip disease. However, postoperative limb length discrepancy (LLD) is a common complication and is reported to be the second most common medical lawsuit in the United States [1–3]. LLD less than 10 mm is now generally considered acceptable [4]. One study noted that 62% of patients experienced an average lengthening of the affected limb of 9 mm after total hip replacement, but only 43% felt true limb lengthening after three months, and only 33% after 12 months [5]. Limp, low back pain and nerve palsy ocur when the LLD is greater than 10 mm. However, there has yet to be a consensus on the upper limit of intolerability for LLD [6].

Currently, to avoid limb length discrepancy after THA, commonly used methods [7] include: (1) Shuck test, (2) drop-kick test, (3) hip stability test, (4) healthy side contrast method. Shuck test is a distraction test to test the soft tissue tension of the hip joint and is generally appropriate with a joint gap of 5 mm. The drop-kick test uses the tension of the quadriceps muscles of the hip and knee to assess the presence of LLD [8]. The healthy side contrast method involves placing both lower limbs symmetrically and determining whether they are equal by touching and comparing anatomical landmarks such as the anterior superior iliac spine, patella, tibial tuberosity or medial ankle. Since LLD is a common complication after THA, various anatomical or reference point marker positioning methods are also used [9, 10]. The use of navigation has also been reported to be effective in reducing LLD [11, 12]. Additional intraoperative x-rays are also a helpful method [13].

However, the above methods are either influenced by the position of the affected limb and have a significant error, or the measurement, although accurate, is cumbersome and expensive to perform.

In this study, a cryo-sterilized paper ruler was used to intraoperatively measure the distance from the anterior superior iliac spine to the tip of the medial ankle compared with the preoperative lower limb length on the healthy side. The method is simple, easy to perform and has high accuracy, and can reduce LLD after total hip arthroplasty. The objective of this retrospective study was to introduce a method of intraoperative measurement to assess the improvement of LLD after total hip arthroplasty and to facilitate clinical promotion.

## Materials and methods

### Patient selection

Patient inclusion criteria: hip joint diseases requiring uncemented total hip arthroplasty after conservative treatment failed, including femoral neck fracture, osteoarthritis, femoral head necrosis, acetabular dysplasia and primary hip joint diseases of indistinguishable aetiology. Exclusion criteria: the presence of compensatory scoliosis and revision patients.

### Patient information

Seventy patients, 27 men and 43 women, average age 59.8 years, range 17–82 years, who received THA from October 2018 to July 2022 according to the above criteria. There were 22 cases of femoral neck fracture, 11 cases of hip osteoarthritis, 24 cases of femoral head necrosis, 4 cases of acetabular dysplasia and 9 cases of primary hip arthrosis of unknown aetiology. There were 22 cases in the fracture group and 48 cases in the non-fracture group.

### Preoperative evaluation and postoperative radiographs

**Fig. 1:**
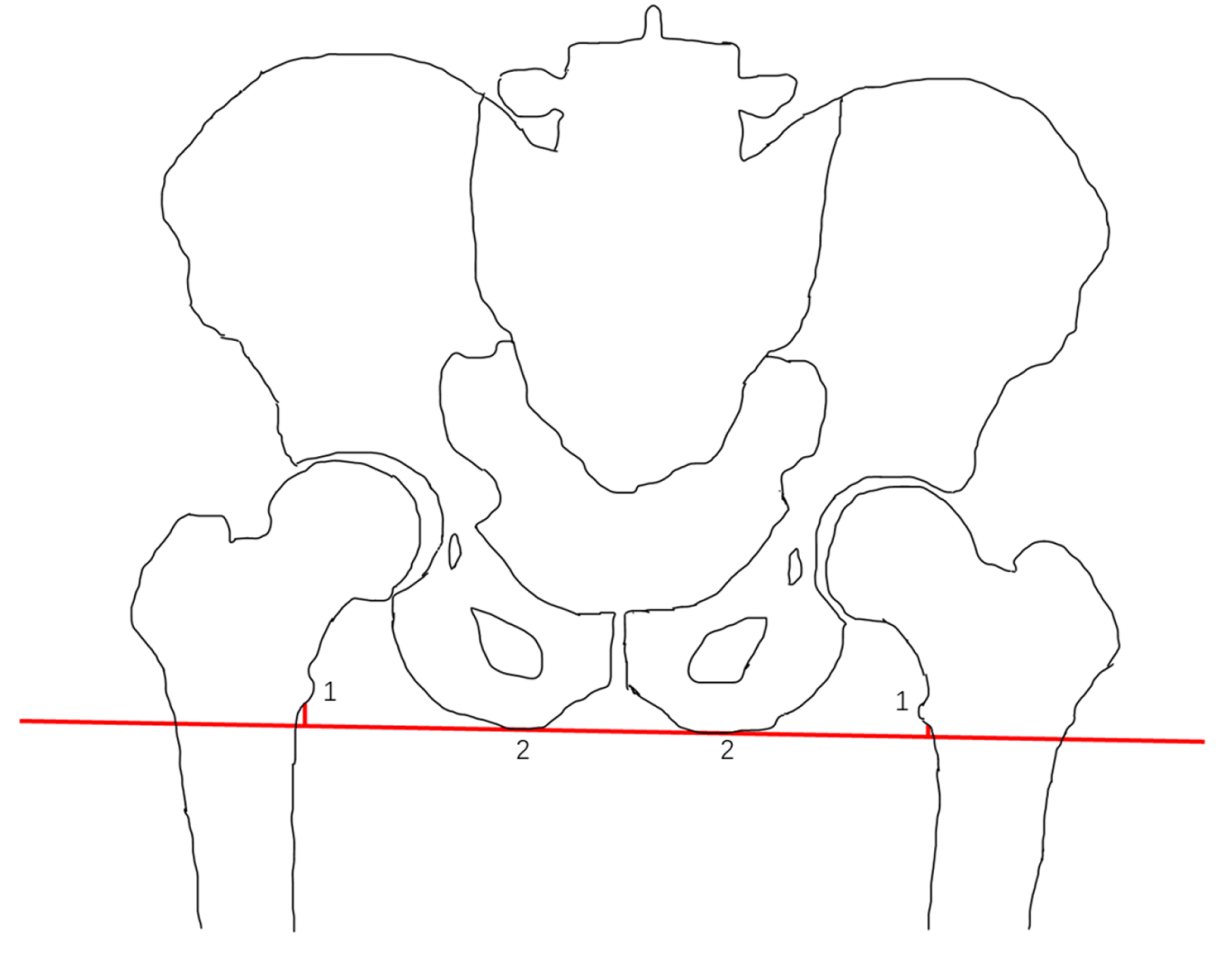
Radiological measurement of LLD in the anterior-posterior pelvic radiograph (1-the lesser trochanter,2- the ischial tuberosity.)

Preoperatively, standard anteroposterior pelvic x-rays were taken to check for shortening of the affected limb, and lumbar x-rays were also taken to rule out lumbar scoliosis. The length of both lower limbs was then measured in vitro and by imaging. Where the length of the healthy limb was measured on the operating table prior to surgery after anaesthesia. In vitro measurement was the distance from the anterior superior iliac spine to the tip of the medial ankle measured bilaterally in the supine position. When performing a standard postoperative anteroposterior pelvic radiograph, the patient’s position was maintained in the standing position with the toes facing forward and the feet kept shoulder-width apart. Radiographic measurement was the perpendicular distance from the lesser trochanter to the line of the sciatic tuberosity on both sides. The difference between the two distances was the length of the inequality of both lower limbs (Fig. 1). A positive value of lower limb inequality indicated a long lower limb length on the replacement side. In contrast, a negative value indicated a short lower limb length on the replacement side. The actual value of lower limb length inequality indicated the degree of lower limb length difference and could be either positive or negative. The absolute value of lower limb length inequality was positive and was used to assess the absolute difference in postoperative LLD. Since the diameter of the acetabular cup was known, the magnification was determined by measuring the diameter of the acetabular cup in the image. With this adjusted magnification, a more accurate bilateral lower limb length difference could be determined. All procedures were performed by the same surgeon using a lateral approach in the lateral position, and all prostheses were provided by DePuy Synthes.

### Surgical method

**Fig. 2:**
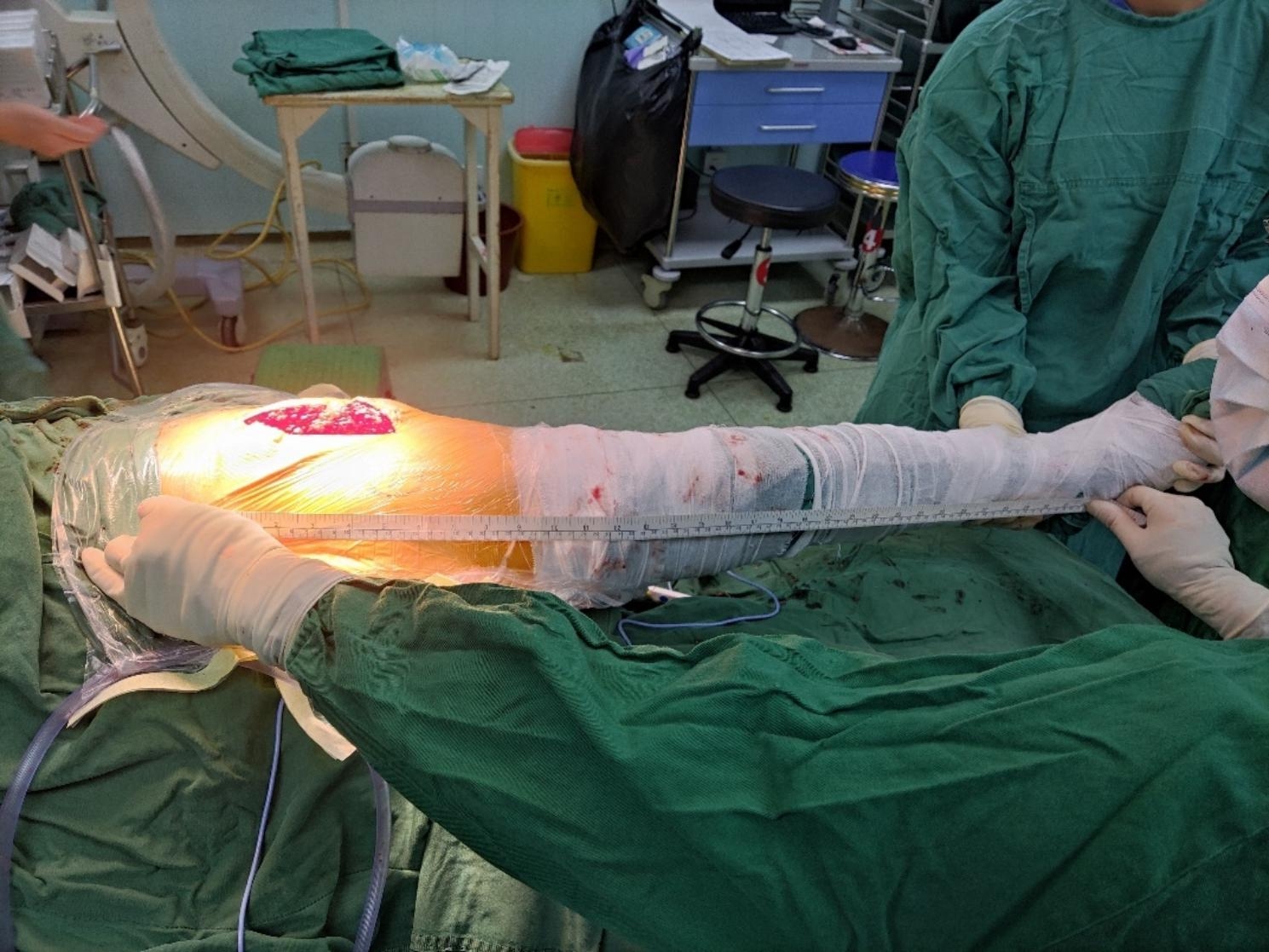
Intraoperative measurement of the lower limb length on the operated side

The patient was placed in the lateral position, and a posterolateral approach was used to separate the gluteus medius and broad fascial tensor bluntly. The anterior part of the hip capsule was incised longitudinally in the direction of the femoral neck. The dislocated hip was osteotomised according to the osteotomy plane of the femoral neck, one transverse finger above the lesser trochanter. The acetabulum was treated and fitted with an acetabular cup and liner. The femoral medullary cavity was then treated, the femoral stem trial mould and femoral head trial mould were installed, and the hip joint was reset. The affected limb was maintained in an abducted neutral position. The distance from the anterior superior iliac spine to the tip of the medial ankle was measured using a cryogenically sterilized paper ruler (Fig. 2). Combining the preoperative measurements of the healthy limb, the femoral head and femoral neck prosthesis were selected by adjusting the depth of the femoral stem and selecting the appropriate size of the femoral head and femoral stem prosthesis. The ideal position for the femoral stem prosthesis was with the stem centered in the femoral canal, and the center of rotation of the femoral head should align with the tip of the greater trochanter. The length of both lower extremities was measured again for comparison, and hip stability was examined in parallel.

### Statistics

The statistics were analyzed by SPSS (Version 23, SPSS Inc., Illinois, USA). Paired t-tests were used to compare preoperative and postoperative measurements. One-sample t-tests were used for postoperative measurements, with a p-value less than 0.05 considered a statistically significant difference.

## Results

The absolute value of postoperative LLD was 6.68 ± 4.48 mm, of which 53 cases (75.7%) were less than or equal to 10 mm, while 30 patients (42.9%) were less than or equal to 5 mm. The actual value of the postoperative LLD was 5.57 ± 6.04 mm, with 56 cases in the affected limb lengthening group (7.66 ± 4.72 mm) and 14 cases in the affected limb shortening group (-2.75 ± 2.46 mm). The absolute value of LLD equal to less than 10 mm was 69.6% in the lengthening group, and 100% in the shortening group (Table 1). Because only the diameter size of the actual acetabular cup was available for correction after surgery, it is not possible to compare the pre- and post-operative scenarios in terms of imaging. Because the absolute values of postoperative LLD conformed to a normal distribution, the difference was statistically significant using a one-sample t-test compared with 10 mm (t = -5.821, p < 0.05).

**Table 1:** Distribution of absolute values of LLD ≤ 10 mm in the lengthening and shortening groups

Group	Total number	Number of absolute values of LLD ≤ 10 mm	Percentage
Lengthening group	56	39	69.6%
Shortening group	14	14	100%

**Fig. 3:**
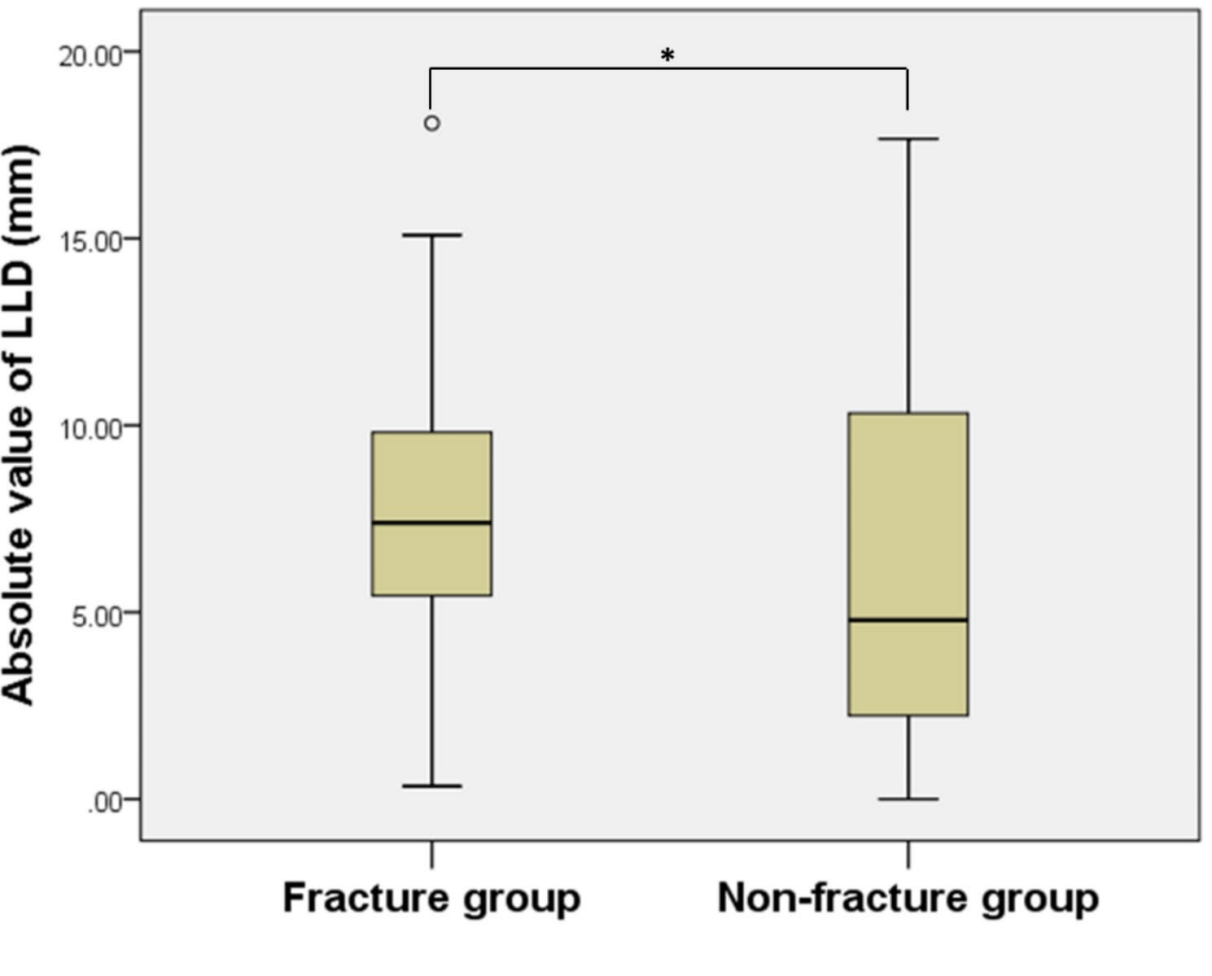
Box plot between fracture group and non-fracture group (The x-axis reveals different groups, and the y-axis reveals the absolute value of LLD. Star (*): p > 0.05)

As total hip arthroplasty was significantly different in the management of the acetabulum in patients with femoral neck fractures and non-femoral neck fractures, which might also result in different LLD, the subjects of this experiment were divided into fracture and non-fracture groups. The absolute value of postoperative LLD was 7.80 ± 4.78 mm in the fracture group compared to 6.16 ± 4.74 mm in the non-fracture group. The difference between the fracture and non-fracture groups was tested using the Mann-Whitney U rank sum test with a p-value equal to 0.117, which was not statistically different (Fig. 3**).**

The absolute value of LLD equal to less than 10 mm was 77.3% in the fracture group, and 75% in the non-fracture group, and the chi-square test did not show a statistically significant difference between them (Table 2)( p > 0.05).

**Table 2:** Distribution of absolute values of LLD ≤ 10 mm in the fracture and non-fracture groups

Group	Total number	Number of absolute values of LLD ≤ 10 mm	Percentage
Fracture group	22	17	77.3%
Non-fracture group	48	36	75%

## Discussion

This experiment used intraoperative measurements of the lower extremity length of the affected limb compared to the preoperative healthy side, which significantly improved the LLD compared to the 10 mm acceptable range. Patients with femoral neck fractures enrolled in this study generally did not have cartilage degeneration of the acetabulum or osteoarthritis of the hip joint [14], and the acetabulum was relatively normal. In contrast, patients with non-femoral neck fractures generally had cartilage degeneration of the acetabulum or osteoarthritis of the hip joint. There were clear differences in the handling of the acetabulum between the fracture and non-fracture groups. On the other hand, grinding the acetabulum could affect LLD to some extent, so it was discussed in two groups. The percentage of postoperative LLD less than 10 mm was 77.3% (7.80 ± 4.78 mm) and 75% (6.16 ± 4.74 mm) in the fracture and nonfracture groups, respectively; however, there was no statistical difference between these two groups. This also suggests to some extent that the method was universally applicable and could be applied to all causes of hip disease.

One study showed that using cemented femoral stems in total hip arthroplasty resulted in a lower leg length discrepancy incidence than non-cemented femoral stems [15]. However, the use of LLD prevention was not described in the study. The use of intraoperative fluoroscopy to prevent LLD in direct anterior approach (DAA) has also been reported, although the authors found no significant difference in postoperative LLD between the fluoroscopic and nonfluoroscopic groups [16]. One review described about 20 different intraoperative techniques to prevent LLD, all of which used a stable reference point in the pelvis and a variable reference point in the femur [17]. Obviously, these techniques also required accurate reproduction of the abduction/adduction position of the femur before and after the trial mould placement. Gupta R et al. used the double-stitch technique to assess LLD in one group and palpation to compare patellar levels in the other. The conclusions suggested that the double-suture technique was a concise and valuable method for reducing LLD after THA [18]. In addition, one investigation used three measurements to compare postoperative leg length discrepancies: direct intraoperative comparison of legs, measurement with a compass-like device fixed above the acetabulum and measurement of the Trochanteric/Joint ratio using an intraoperative device. The last method produced the best results, with approximately 85% of participants having a postoperative LLD of less than 5 mm [19]. However, this method required special devices designed by the prosthesis company and was not universally applicable.The two lines used in this experiment to measure LLD were the ischial tuberosity line and the lesser trochanter line. A study was conducted to measure leg length discrepancy (LLD) using four lines: the acetabular teardrops, the ischial tuberosity, the sacroiliac joint, and the foramen occulta. The measurement was taken from these lines to the lesser and greater trochanters. The study found that the most accurate measurement was from the line connecting the ischial tuberosity to the lesser trochanter [20]. For measuring LLD on pelvic radiographs, this study used the difference in distance between the bilateral ischial line and the bilateral lesser trochanteric line. One investigation showed that using teardrops and femoral centres was more accurate [21]; however, identifying teardrops and femoral centres on radiographs was not easy.

Although intraoperative reliance on palpation of the anterior superior iliac spine and medial ankle under drapes was not very accurate [22], preoperative and intraoperative measurements were taken by the primary surgeon, and multiple measurements were taken to minimize errors. The advantages of this method, which are simple to operate, easy to implement, and less time-consuming, are worth promoting.

The postoperative LLD was not measured clinically in this experiment, as it has been reported that clinical and radiological measurements do not correlate significantly [23, 24]. When LLD was calculated on anteroposterior pelvic films, there was some error. A study utilizing full-length standing anteroposterior radiographs measured bilateral leg length, femur length, and tibia length. The results showed that around 1/6 of patients exhibited a leg length discrepancy (LLD) exceeding 10 mm when measured from the lesser trochanter [25]. It was also shown that the average magnification for limb length measurement using Computed Radiography (CR) over EOS imaging was 6.8%[26]. In addition, it was found that conventional measurements of LLD on AP pelvic radiographs did not correlate well, while the full-length, functional imaging EOS method could provide a better evaluation of LLD [27]. However, EOS is not standard for all hospitals and lacks universal applicability. On the other hand, deviation from the standard anterior-posterior pelvic position could impact the measurement of leg length discrepancy (LLD). One study showed that when the pelvis was rotated more than 20 degrees, it affected the measurement of pelvis-related parameters [28]. In addition, it was reported that a 5-degree femoral abduction/adduction misalignment could significantly change leg length by 8 mm [29]. Because the leg might be in a different rotational position when the X-ray was taken, the accuracy of measuring LLD was relatively poor compared to CT scan images [30, 31]. Nevertheless, CT radiation was relatively high and was not a routine examination for THA.

In this experiment, the preoperative measurements and intraoperative measurements were taken with the femur in an abducted neutral position on the measuring side, thus reducing the effect of femoral position on the measurement results. However, femoral positions influenced the method of measuring lower limb length in this study. It was important to note that during surgery, there was no guarantee that the position of the affected side measured intraoperatively would match the position of the healthy side measured preoperatively in this study. This was one of the limitations of this study. This study did not clinically compare the differences in preoperative and postoperative LLD, which was its deficiency and required further improvement. Meanwhile, this study did not elucidate the effect of LLD on function and gait, and further studies are needed in the future.

## Conclusions

The intraoperative method of measuring the length of the lower limb using a paper ruler compared with the length of the preoperative healthy lower limb is simple to operate and can effectively reduce postoperative LLD.

## Data Availability

The data collected in this study will be stored in our hospital permanently. The data used or analysed during the current study are available from the corresponding author on reasonable request.
